# Optimization of Quantum Dot Thin Films using Electrohydrodynamic Jet Spraying for Solution-Processed Quantum Dot Light-Emitting Diodes

**DOI:** 10.1038/s41598-019-50181-5

**Published:** 2019-09-25

**Authors:** Tuan Canh Nguyen, Thi Thu Thuy Can, Woon-Seop Choi

**Affiliations:** Department of Display Engineering, Hoseo University, Asan, Chungnam 31499 Korea

**Keywords:** Nanoparticles, Quantum dots

## Abstract

The electrohydrodynamic (EHD) jet spraying process is a good method for making quantum dot (QD) layers in light-emitting diodes (LEDs). However, controlling the morphology and large-scale fabrication of the QD layers are critical for realizing all-solution-processed QD-LEDs with high performance. Three spraying techniques were used with the EHD jet spraying technique: a big circular film method, a spiral-line method, and a straight-line method. These techniques were used to obtain QD films with good uniformity. The straight-line spray showed the most promise to obtain a uniform QD layer with large area, and QD-LEDs made with this method showed better performance with a low turn-on voltage of 3.0 V, a luminance of 7801 cd/m^2^, and a maximum current efficiency of 2.93 cd/A.

## Introduction

Nanoparticles with dimensions in the range of 1–20 nm are called quantum dots (QDs). Zero-dimension QDs are described as artificial atoms because of their 𝛿-function-like density of quantum states, which can lead to narrow optical line spectra. The size-dependent electrical and optical properties of QDs can be exploited in unusual classes of electronics and optoelectronic devices with potential for use in information displays, solid-state lighting, biology, and other systems^1^.

Colloidal QDs are of interest for display devices for several reasons, including high quantum yields, low photo-bleaching, absorbance, size-controlled emission, narrow emission peaks, and compatibility with solution processes^2^. QD light-emitting diodes (QD-LEDs) are a form of light-emitting technology that consists of nano-scale crystals, which can provide an alternative for applications in display technology. The structure and behavior of QD-LEDs are very similar to those of OLEDs, but the emitter in QD-LEDs is semiconductor nanoparticles, which are typically deposited between the charge carrier layers^3^.

Conventional vacuum deposition is used to fabricate OLEDs but is not suitable for heavy metal materials like CdSe/ZnS core/shell QDs. Spin coating has been the most popular approach to fabricate QD-LED devices, mainly due to the simplicity of the method for achieving a near-monolayer of nanoparticles. However, the method results in a relatively high cost of QDs and the loss of more than 96% of the solution during spin coating. Therefore, an alternative deposition route that is capable of patterning and consuming a minimum amount of QD materials is needed.

QD chemistry seems rather simple, but obtaining a sufficiently small size distribution is an enormous challenge^4^. Achieving this is costly and laborious and, is also the main cost factor in QD-LED manufacturing. Therefore, device fabrication should be as simple as possible, and electrohydrodynamic (EHD) jet printing may be an alternative solution^5,6^. EHD jet printing is a robust, simple, flexible, inexpensive, and powerful technique for a wide range of micro- and nano-particulate patterning and thin-film deposition processes^7,8^.

Recently, EHD jet printing has been used to prepare QD thin films for solid-state lighting devices. A QD/polymer nanohybrid thin film was fabricated and applied on top of blue LEDs to obtain W-LEDs^9^. CdSe/ZnS core/shell QDs were employed as the emissive layer in QD-LED devices, but resulted in low-performance devices with a maximum luminance of 2.2 lux at 15 V^10^. EHD jet printing was used with a simultaneous program for moving the substrate to fabricate an array of QD droplets. Even though high-performance red and blue QD-LEDs were obtained, this method is only suitable for printing on a very small area, and it is difficult to obtain a smooth thin film on large substrates^11^.

Due to the simplicity and versatility of EHD jet printing, it has been successfully applied to obtain particulate materials with controllable configurations, morphologies, structures, thicknesses, sizes, and shapes. The primary benefit of EHD jet printing is atomization in the Taylor cone-jet mode (also known as electrospray deposition), which can be applied to create a thin film on various substrates. Using EHD jet printing, the thickness and morphology of QD patterning can be controlled through the combination of printing parameters, such as the size of the nozzle, tip height, injection rate, voltage bias, and spraying time^12,13^.

However, the most common problem in the spray coating technique is high roughness of the coating surface due to large spray particles. Large spray particles cannot be applied to nano-thin film coatings for printed electronic devices. Generally, high variation in the coating thickness leads to poor device performance^7,14,15^. Furthermore, EHD jet printing is suitable for only small areas, and it is difficult to apply on a large scale to obtain uniform and smooth thin films for use in QD-LED manufacturing^16^. Hence, we propose three unique spraying techniques to fabricate large-scale QD thin films instead of printing. With these methods, various morphological surfaces of large QD patterns were made with EHD jet spraying to obtain an emitting layer for QD-LEDs that are all solution-processed except for the electrodes.

## Results and Discussion

**Figure 1:**
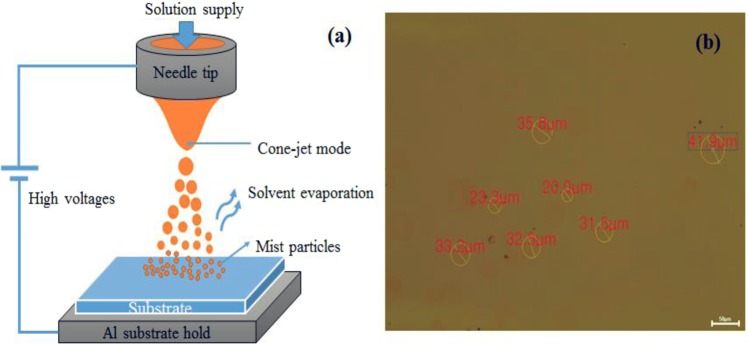
(**a**) Cone-jet mode of EHD jet spraying, (**b**) OM image of separate QD droplets on ITO glass at a flow rate of 0.016 μl/sec, tip height of 12 mm, and applied voltage of 3.8 kV.

To make large size and uniform QD films, instead of EHD jet printing, EHD jet spraying was employed to generate micro-QD droplets from solution injection in a capillary, where a high voltage is applied to a single metallic nozzle, as shown in Fig. 1a. In order to apply the droplets on a QD-LED multilayer, it is necessary to obtain smooth QD thin films that can cover all surfaces of ITO glass patterns (with an area of 1 in. x 1 in.). During EHD jet spraying, micro-droplets can be generated as mist particles in cone-jet mode from a solution injected into a metallic nozzle by applying a high voltage^6,17^. The mist is then drawn toward the substrate, and the hexane solvent instantly evaporates, leaving QDs on the surface, as shown in Fig. 1b.

**Figure 2:**
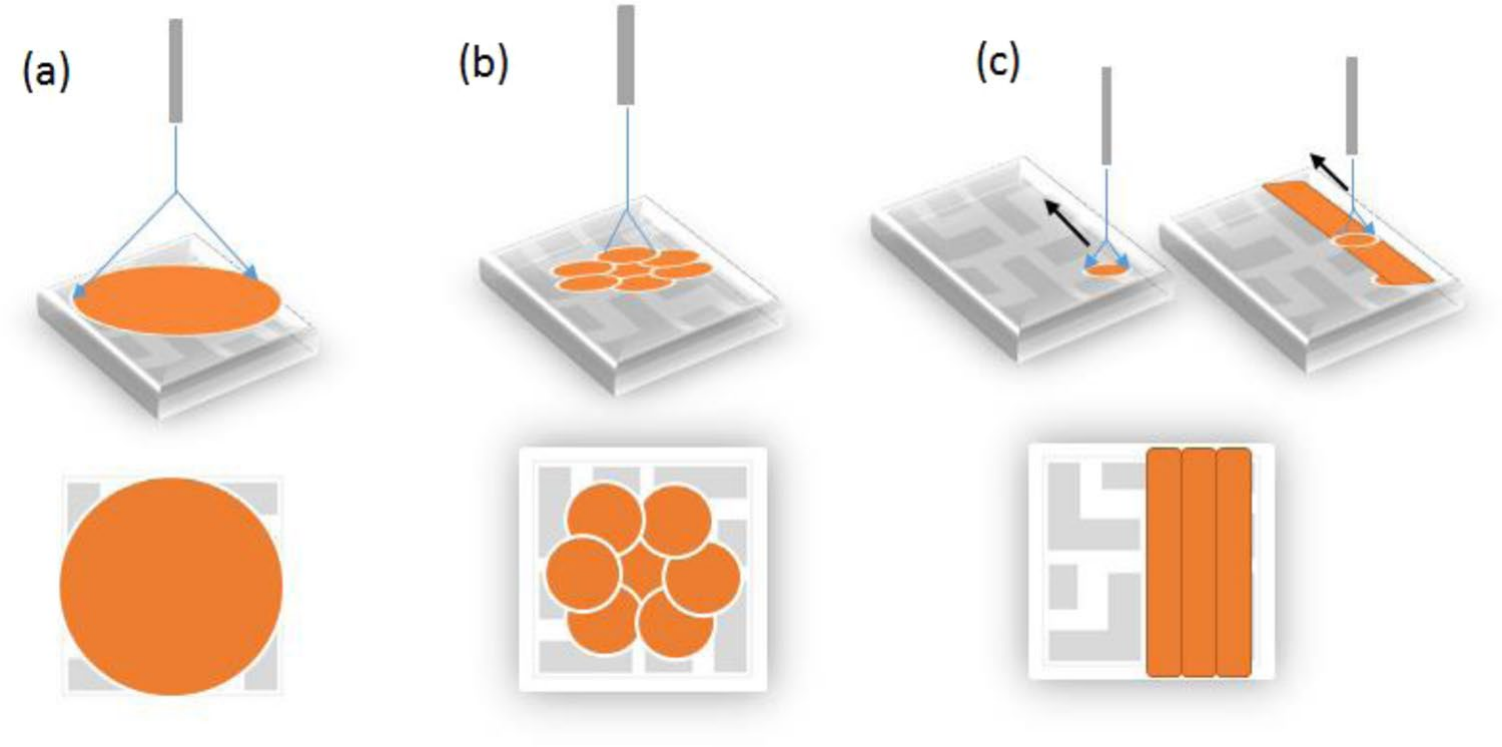
Three types of EHD jet spraying methods; (**a**) big circular thin film, (**b**) spiral line, and (**c**) straight line methods.

The size and morphology of printed circular QD patterns depend on the standoff distance (tip height) and the applied voltage. The overlapping of droplets causes differences in the thickness and morphology between the center and surrounding areas of circular patterns. Therefore, to obtain large, uniform, smooth QD thin films, we present spraying methods based on various adjustments of the EHD jet parameters, the tip height, and the moving substrate, as shown in Fig. 2.

Changing the power of the EHD jet printing affects the morphology of the jetting modes and determines the stability of the cone-jet mode. Multiple-cone spraying mode also takes place. Shear stress occurs on the liquid surface of the QD solution due to the established electric field, which causes elongation of the jet and disintegration of the liquid into a mist of small QD droplets. The electric field strength at the end of the capillary can be calculated using the following equation^18^.

$${E}_{c}=\frac{2{V}_{c}}{{r}_{c}ln(4d/{r}_{c})}$$

where V_c_ is the input voltage, r_c_ is the outer diameter of the capillary tip, and d is the distance between the capillary tip and the grounded substrate.

When the applied voltage is high or the outer diameter of the capillary tip is small, the electric field strength becomes larger. This large strength generates small droplets from the injected solution. The flow rate was maintained at 0.016 µl/sec, the distance between the capillary tip and substrate was 25 mm, and the outer diameter of the capillary tip was 0.23 mm. In the range of 2.8–4.9 kV, the jetting mode of the systems is cone-jet mode. At other values, the jetting mode can be dripping mode, micro-dripping mode, oscillating-jet mode, or multi-jet mode. At a high voltage, it is possible to generate smaller QD droplets that are directed to the substrate, and cone-jet mode was maintained in the spraying process due to stable jetting.

Hexane was chosen as a solvent due to its low boiling point (about 69 °C) and to minimize the damage in the underlying layer. However, n-hexane evaporates quickly and has low viscosity, which affects the stability of the Taylor cone-jet mode. In the EHD jet spraying process, the QD hexane liquid droplets are pulled out from a liquid thread, spread out, and touch the substrate, resulting in a diverse collection of rough droplet marks. Various parameters and spraying methods affect the drop formation, including the size, shape, type, and morphology of single droplets.

A thin film made by the EHD process is a collection of mist particles. The size of the mist particles directly determines the size of the droplets on the substrates. It is not easy to find the exact size of mist particles during the process. However, the diameter of QD droplets on ITO glass is approximately 30 μm when using a flow rate of 0.016 μl/sec, tip height of 12 mm, and applied voltage of 3.8 kV. Figure 1b shows an optical microscope (OM) image of separate QD droplets on ITO glass and the remaining QDs after solvent evaporation. The consolidation mechanism of the EHD-sprayed droplets could be similar to that of spray drying. The difference is that EHD-sprayed droplets are usually smaller and have charges on the surface, resulting in enhancement of the evaporation rate of the solvent in the spraying process.

**Figure 3:**
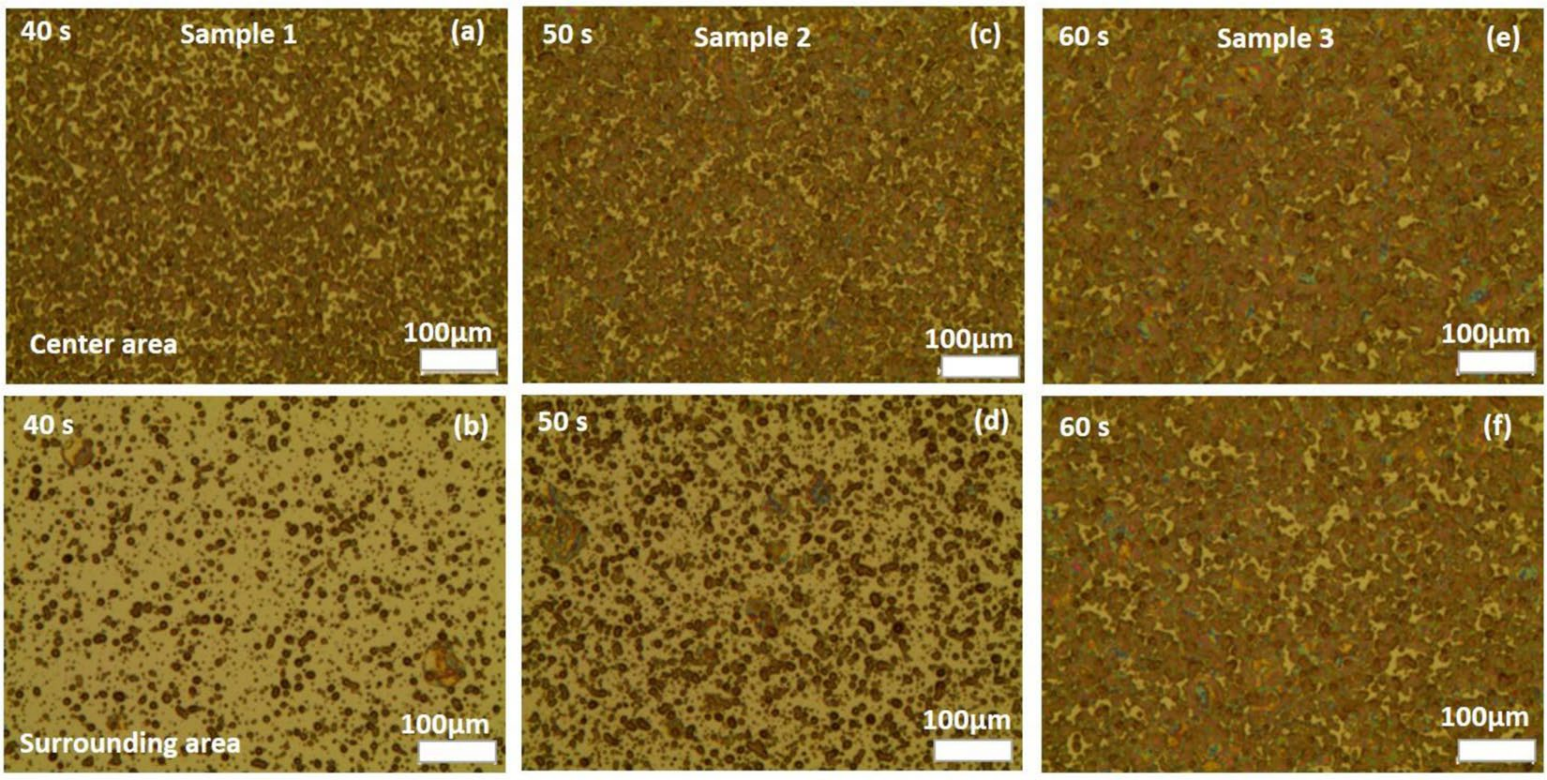
OM images of QDs on ITO glass using type I spraying method at an applied voltage of 4.9 kV, tip height of 55 mm, flow rate of 0.016 µl/sec, and spraying time of 40–60 s.

Figure 3 shows OM images of the QD patterns formed by the type I spraying method in Fig. 2a. In this method, the voltage was kept at 4.9 kV to obtain a stable cone-jet mode. The height of the metallic tip was kept at 5.5 cm to obtain a large circular QD thin film that covered whole ITO surface pattern. Cone-jet mode also was stable at flow rates of 0.008 to 0.024 µl/sec. In this type of spraying, the flow rate was kept at 0.016 µl/sec, and the morphology of the QD thin films depended on the deposition time in the range of 40–60 s. By changing this factor, the droplet density and thickness on the two main areas of the QD patterns is also changed. In this case, a high standoff distance and input voltage affect the size of QD droplets, but it is less correlated with the higher of tip height.

The density of droplets is higher in center areas than in the surrounding areas (Fig. 3). The thickness of the sample was approximately 280 nm at the center of the circular QD pattern deposited for 40 s, but it was just 60 nm in the surrounding area. The difference in thickness of the areas on the same sample causes defects on the surface of the next layer (ZnO), which is deposited by spin coating in QD-LED devices. Therefore, the QD-LED devices will have low performance. The optical and electrical properties of the QD-LEDs are shown in Table 1, and a maximum luminance of 1449 cd/m^2^ was obtained with a spraying time of 50 s.

**Table 1:** Optical and electrical properties of QD-LED devices using type I spraying method (big circular thin film) with various conditions of EHD jet spraying.

Time (sec)	Max. LE (cd/A)	EQE (%)	Max. Luminance (cd/m^2^)
40	0.449256	0.5352	844
50	0.610383	0.3536	1449
60	1.025466	0.4325	1226

To solve the problem of the different thicknesses between the two areas in the type I spraying method, we present another way to obtain large uniform QD patterns by reducing the size of the circular QD films, as shown in Fig. 2b. In this method, the applied power was maintained at 3.3–3.9 kV, and the flow rate was in the range of 0.016–0.032 µl/sec to obtain a stable cone-jet mode. The standoff distance was kept at 25 mm to obtain a small, uniform circular QD thin film of 8 mm diameter. Moving the substrate along a spiral line shape resulted in a collection of small and circular thin films. The spraying time was approximately 3–4 s to obtain a suitable thickness around 120 nm. The spiral-line spraying method (type II) produced a large QD thin film, which was a collection of small, uniform, circular thin films with overlapping areas. Changing the moving distance of the ground substrate (XY-stage distance) is the main factor in this method.

**Figure 4:**
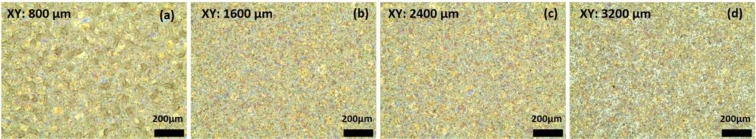
OM images of QDs sprayed on ITO glass using type II spraying method at an applied power of 3.6 kV, tip height of 25 mm, flow rate of 0.032 µl/sec, and changing XY-stage from 800 to 3200 µm.

Figure 4 shows OM images of the QD thin films on ITO glass when the (X, Y) coordinates were changed from (800 µm, 800 µm) to (3200 µm, 3200 µm). The overlapping areas at (800 µm, 800 µm) are much larger than at (3200 µm, 3200 µm), which corresponds to the roughness of the surfaces and a low density of QD particles on the thin film. Understanding the correlations between the morphological nanostructure and the photocurrent generation is desirable for improving the device performance. Table 2 shows that the luminance was 454.5 cd/m^2^ at (800 µm, 800 µm) and increased to a maximum of 1780 cd/m^2^ at (2400, 2400 µm).

**Table 2:** Optical and electrical properties of QD-LED devices using type II spraying method (spiral line) with various conditions of EHD jet spraying.

X (µm)	Y (µm)	Max. LE (cd/A)	EQE (%)	Max. Luminance (cd/m^2^)
800	800	0.572025	0.306	454.5
1,600	1,600	0.875517	0.4737	739.8
2,400	2,400	0.85085	0.5228	1780
3,200	3,200	1.851119	1.441	1118

QD-LED devices fabricated by the type II method had better performance than those made with type I, but the overlap areas remaining between the circular thin films were still a challenge to solve. Therefore, Fig. 2c shows the straight-line (type III) spraying method to obtain better QD thin films. The spraying method is a combination of moving the substrate while spraying in certain directions. To obtain a large thin film, the flow rate, applied voltage, and working space are kept constant, while the XY-stage is moved at 2000 μm/s. The substrate was taken along one direction (Y) after another direction (X) of the horizontal plate, and this process was repeated.

In order to obtain a stable cone-jet mode, the applied voltage was kept at 3.6–4.8 kV. The standoff distance was maintained at 12 mm to obtain small circular QD thin films with a diameter of 3.4 mm. The two main factors that determine the uniform ring droplets in this method are the rapid movement of the substrate from (800 µm, 800 µm) to (3200 µm, 3200 µm) along straight lines and changing of the flow rate from 0.016 to 0.032 µl/sec.

**Figure 5:**
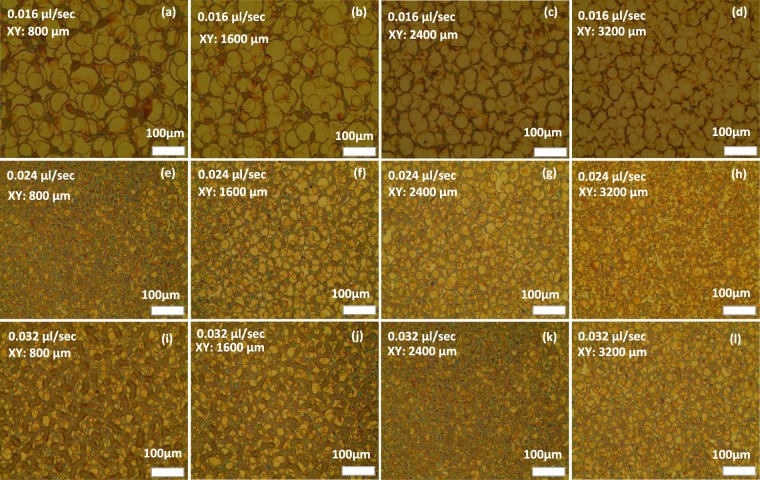
OM images of QDs sprayed on ITO glass using type III spraying method at an applied voltage of 3.8 kV, tip height of 12 mm, and changing XY-stage in range of (800, 3200) µm, and the flow rate from 0.016 to 0.032 µl/sec.

Figure 5 shows the surface morphology of single droplets and the continuous thin film. There seems to be no difference in the thickness on all large-area QD patterns, and there are no overlapping areas of small circular thin films in the thin continuous films. Controlling the size, shape, and roughness of the QD droplet boundaries is the most important in this method.

The configuration of the single droplet residues is essential for the morphology of the QD thin film and the electrical/optical characteristics of the QD-LED devices. In order to study the morphological changes in different process conditions, 12 QD films were prepared at three flow rates combined with four substrate stage movements. The OM images in Fig. 5 show the variation of the circular residue sizes and shapes for different combinations of flow rates and XY distances.

The droplet residue resembles a dish with a thick rim. Such residue shapes are often called “coffee rings”. The evaporation rate of n-hexane is higher at the contact line, and there is continuous inflow of liquid. As a result, the dried region gradually accumulates, and the central area drains to form the coffee-ring shape. The diameter of the droplets changed from 1 µm (Fig. 5h) to 40 µm (Fig. 5d), and a uniform of 10 µm was obtained (Fig. 5f,g,l). At a suitable flow rate, the boundaries between the circular residues are ambiguous, and more continuous films are formed. The form of the boundaries and the surface roughness of the thin films are consistent with the rim of the single droplet residue, as demonstrated in Fig. 5a–d in comparison with Fig. 5e–h. However, when the flow rate is too high, the ring structure of droplets gradually disappears, and the surface becomes rugged (Fig. 5i,j).

The nanostructures of the bulk inside in the spin-coated films are naturally homogeneous. However, the bulk nanostructures in the films deposited with the EHD jet spraying are dependent on the piling up of droplets during the spraying process. The colors in Fig. 5e–h show the thickness differences and morphology of the samples from 3D images from the NanoSystem in Fig. S1 in the Supplementary Information (S.I.). The SEM results of samples fabricated at a flow rate of 0.016 µl/sec are shown in Fig. S2 in the S.I. The rugged boundaries of rings are clearly shown in Fig. S2a in the S.I. Fig. S2b illustrates the uniformity of tiny QDs at the centers of the droplets.

The thickness and roughness of the QD films are important factors for the device performance. The film surface becomes smoother and uniform with a flow rate of 0.024 µl/sec and coordinates of (2400 µm, 2400 µm) (Fig. 5g), resulting in a maximum luminance of 7801 cd/m^2^ (Table 3). In these conditions, the average thickness of the QD thin film on the ITO glass is 110 nm. The root mean square (RMS) roughness was 0.0492 µm. In the same conditions, the RMS roughness of the QD patterns sprayed on top of the PVK/PEDOT:PSS/ITO-glass was 0.0564 µm. The other roughness values in the different conditions are shown details in Table S1 in the S.I.

**Table 3:** Optical and electrical properties of 12 QD-LED devices using type III spraying method (straight line) with various conditions of EHD jet spraying.

Flow rate (µl/ sec)	X (µm)	Y (µm)	Max. LE (cd/A)	EQE (%)	Max. Luminance (cd/m^2^)
0.016	800	800	0.59333	0.2771	1102
	1600	1600	1.91154	0.9958	3994
	2400	2400	1.227381	0.6205	2286
	3200	3200	0.963395	0.3814	738
0.024	800	800	2.939027	1.4067	5690
	1600	1600	0.968192	0.6352	6374
	2400	2400	1.365715	0.9099	7801
	3200	3200	0.459512	0.3215	4114
0.032	800	800	0.523012	0.4107	2906
	1600	1600	0.593401	0.4764	3072
	2400	2400	1.357839	0.894	6302
	3200	3200	0.576508	0.4696	5209

The ruggedness of the surface of QD patterns also affected the various surface defects of the next spin-coated layer and cathode, resulting in poor electro-optical properties of the QD-LED devices. As shown in Fig. 5i, at the same flow rate of 0.032 µl/sec, the substrate movement at a short distance (X and Y by 800 µm) caused many overlaps between QD droplets, and the ruggedness of the emission layer resulted in a luminance of 2906 cd/m^2^. Moreover, at the minimum flow rate of 0.016 µl/sec and the largest movement distance (X and Y by 3200 µm), Fig. 5d clearly shows low-density QD materials with ambiguous QD droplets. Consequently, less emission materials in the QD-LED multilayer structure result in a minimum luminance of 738 cd/m^2^.

**Figure 6:**
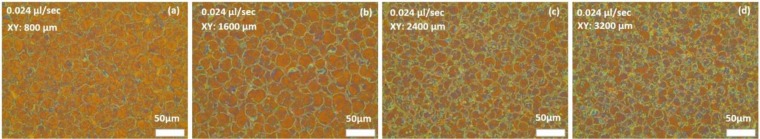
OM images of QDs sprayed on PVK/PEDOT:PSS/ITO-glass patterns using type III spraying method (corresponding to Fig. 5e–h).

**Figure 7:**
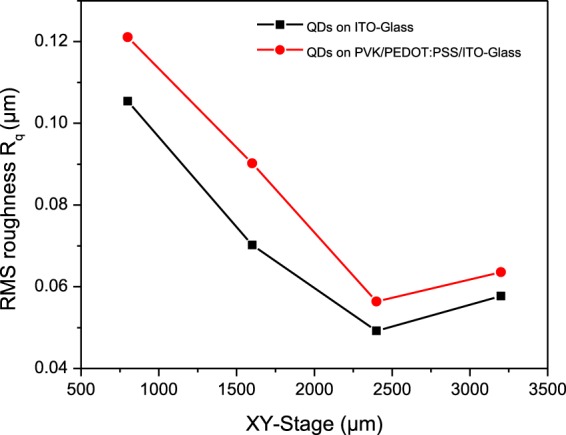
RMS roughness of QD thin films on ITO glass (corresponding to Fig. 5e–h) and QD thin films on PVK patterns (corresponding to Fig. 6).

Figure 6 shows an OM images of the sprayed QD layer on top of the PVK/PEDOT:PSS/ITO-GLASS under the following conditions: flow rate of 0.024 µl/sec, (X,Y) changed from (800, 800 µm) to (3200, 3200 µm), tip height of 12 mm, applied voltage of 3.8 kV, and using the type III spraying method. As shown in Fig. 7, the RMS roughness of QD-sprayed on the PVK/PEDOT:PSS/ITO-GLASS was compared with the RMS of QD-sprayed on ITO glass (Fig. 5e–h). The roughness of the QD-sprayed on ITO glass with a starting RMS roughness of 0.1054 µm was examined as a function of overlapping.

In the same conditions, the RMS roughness of the QD-sprayed on top of the PVK/PEDOT:PSS/ITO-glass was a little higher at 0.1211 µm, which is a consequence of spraying on multiple layers. The minimum RMS of the large QD thin film sprayed on ITO glass was 0.0492 µm when XY was increased to (2400, 2400 µm). However, the RMS was increased to 0.0577 µm by moving substrate at XY (3200, 3200 µm), indicating more roughness due to the difference in overlapping areas between small circular QD thin films, as shown in Fig. S3 in the S.I. A similar linear graph of two RMS roughness groups also indicated the effectiveness of the sprayed QD morphology.

**Figure 8:**
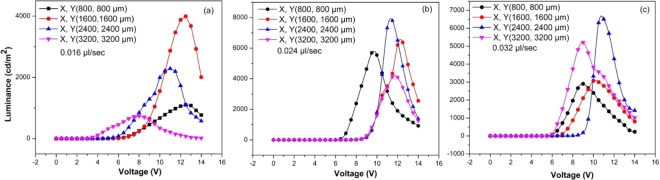
J-V-L graph of 12 QD-LED devices using type III spraying method with various X, Y and flow rates.

A total of 12 QD patterns were sprayed on top of PVK/PEDOT:PSS/ITO-glass while changing the flow rate from 0.016 to 0.032 µl/sec and (X,Y) in the range of (800 µm, 2400 µm). The detailed electro-optical characteristics of the 12 QD-LED samples are summarized in Table 3. The QD layers were sprayed using the type III spraying method on 1 in. × 1 in. substrates. Figure 8 shows the current density-luminance-voltage (J-L-V) characteristics of the sprayed red QD-LED devices based on a normal structure. The low turn-on voltage at 2.5 V in Fig. 8a indicates efficient carrier injection, which could lead to low operating voltage and power consumption.

Fig. S4 in the S.I. shows the QD-LEDs lifetime measurement for the device with the highest luminance, which had an initial luminance of 500 cd/m^2^ and 120 cd/m^2^ under ambient conditions (22 °C and a relative humidity of 20%). With the initial luminance of 500 cd/m^2^ and current of 40.02 mA, the luminance of the sample dramatically decreases to approximately 150.2 cd/m^2^ after one day. With an initial luminance of 120 cd/m^2^ and current of 24.56 mA, the device exhibits a longer half-life time of 4.5 days (around 110 hours) without encapsulation. These results show satisfactory performance compared with other QD-LED devices^19^.

**Figure 9:**
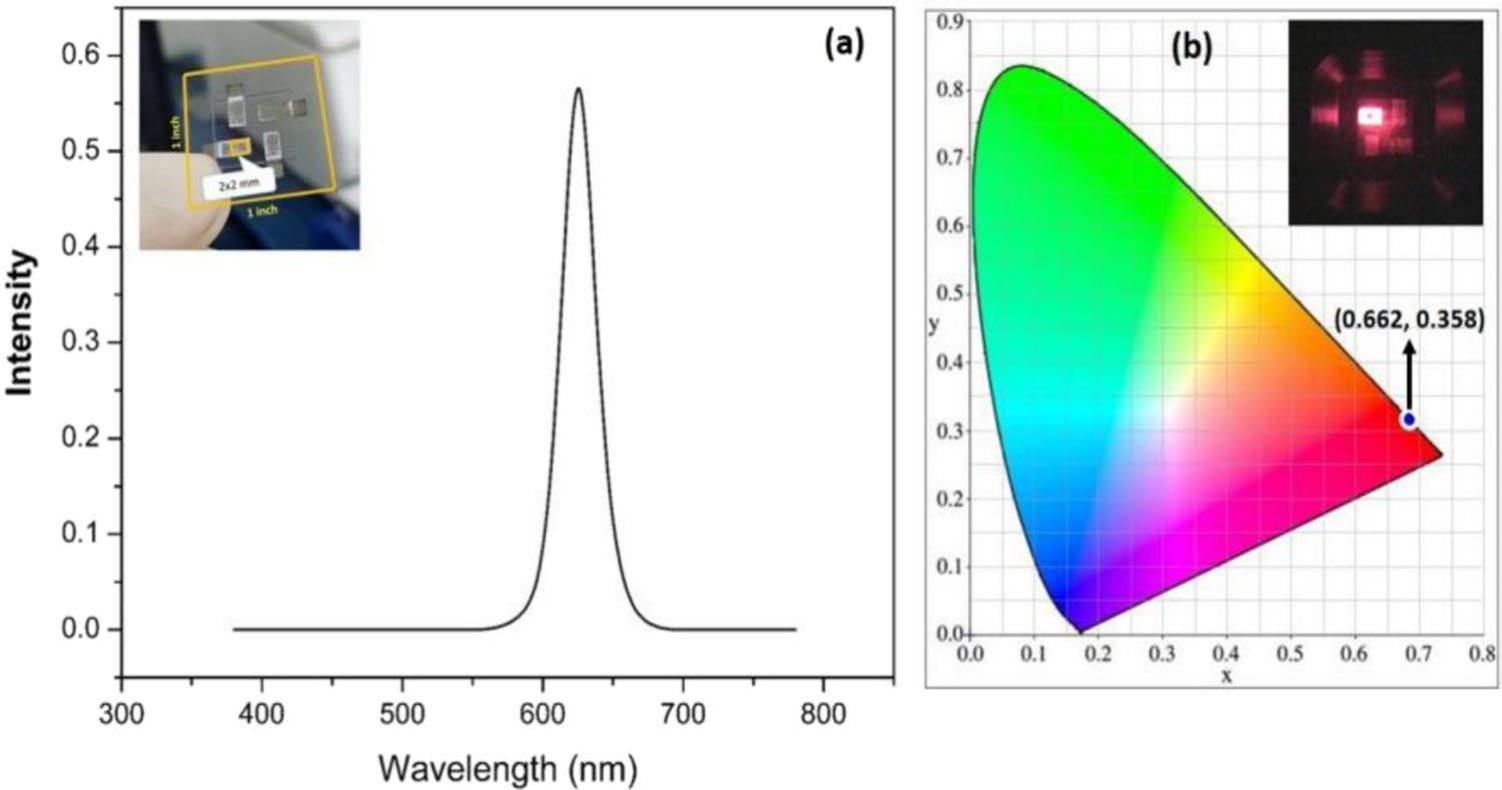
(**a**) EL, Inset: picture of a real QD-LED device, (**b**) CIE, Inset: picture of an illuminated device.

Figure 9a shows the EL properties. There is a sharp emission peak at 623 nm, which is due to colloidal QDs. A square cathode (2 × 2 mm^2^) defined the active area of the QD-LED devices, as shown in the inset. The Commission Internationale de l'éclairage (CIE) chromaticity diagram revealed coordinates of (0.662, 0.358) with a saturated deep-red color, as illustrated in Fig. 9b. The inset clearly shows the illuminance image of the QD-LED device measured by the J-V-L characterization systems.

## Conclusions

In conclusion, we demonstrated large-area patterns of QDs and the fabrication of QD-LEDs using EHD jet spraying. This fascinating and powerful tool has many benefits, such as simple and versatile control, low cost, large-scale, and compatibility with multilayer-structure electronic devices like QD-LEDs. Among the three spraying techniques, a straight-line was the most effective and adaptable. This method obtained a smooth, uniform QD layer with nano-scale thickness (110 nm), after spraying tiny QD droplets of 10-µm diameter). Using this spraying technique, a QD-LED was successfully fabricated and demonstrated a maximum of luminance of 7801 cd/m^2^, a maximum current efficiency of 2.93 cd/A, a maximum EQE of 1.41%, and a narrow full-width. We believe that EHD jet printing will pave the way for the fabrication of multilayer-structure devices and allow for the use of various solvents in a direct patterning method that is convenient and optimal for commercial production.

## Methods

### Material preparation

A QD solution was prepared by mixing 1 ml of red CdSe/CdS/ZnS colloidal QDs (50 mg/ml, Ecolumy Co., Ltd.) with 4 ml of hexane to achieve a concentration of 10 mg/ml, followed by sonication for 10 min. The ZnO nanoparticles were synthesized according to a previous report^20^. Zinc acetate dihydrate (1.18 g) was dissolved into a flask with methanol (50 ml) under vigorous stirring at 65 °C. A solution of 0.592 g KOH in 26 mL methanol was then added dropwise to the flask at 65 °C over a period of 15 min. The mixture was stirred at 1000 rpm for 2.5 h.

After cooling to room temperature, the supernatant liquid was poured out, and the precipitate was washed three times with 20 mL methanol using a centrifuge at 3200 rpm to obtain ZnO nanoparticles (NP). A 4-mL solution of n-Butanol, methanol, and chloroform (volume ratio of 14:2:2) was added to disperse the precipitate and produce a ZnO NP solution with a concentration of 6 mg/ml. The ZnO NP was filtered before using a 0.45-µm PTFE-H syringe filter. Poly (9-vinylcarbazole) (PVK, 0.05 g) was dissolved in 5 ml of chlorobenzene to prepare PVK solution. The solution was stirred for 2 h at 500 rpm and room temperature.

### Preparation of EHD jet printing process

A diagram of the EHD jet printing process is shown in Fig. S5 in the S.I. The method uses a motorized pump, a syringe with a plastic tube, a metal needle as a nozzle, a high-voltage power source, a grounded substrate as a collector, and a computer connected to a high-speed camera. The moving of the grounded substrate was controlled by a motorized aluminum substrate.

The entire process was interfaced with a computer and monitored by software with a high-speed camera. The QD solution was ejected through a metallic tip (I.D. 0.10 mm; O.D. 0.23 mm) at a specific flow rate using the syringe pump. The surface morphology of the QD patterns depends on numerous components and parameters: the applied voltage pulse, pulse frequency, size and shape of the metallic tip, tip height, flow rate, viscosity and conductivity of the solution, rate of solvent evaporation, falling speed of the droplets, spraying time, and speed and direction of the moving collector.

The electric power was in the range of 2.8–4.9 kV. The height of the nozzle tip (Z-axis), distance of the step moving collector (XY-stage), spraying time; and injection rate were changed to identify suitable parameters. The surface morphology was observed using a scanning electron microscope (SEM; SNE 4500 M), optical microscope (OM; Olympus BX51M), and 3D profiler (NanoSystems NV- 2000).

### Fabrication of QD-LED devices and characterization

Fig. S6 in the S.I. shows the regular device structure of the ITO/PEDOT: PSS/PVK/QDs/ZnO/Al layers with an energy band gap diagram^21^. All devices were fabricated on ITO-patterned glass (20 Ω/□), which was subjected to a cleaning process with isopropyl alcohol/acetone under ultra-sonication for 20 min, and then UV-ozone treatment for 20 min.

A hole injection layer (HIL, PEDOT:PSS (Clevios P VP AI 4083)) was spin coated onto the substrate at 3500 rpm for 60 s and annealed at 150 °C for 30 min. In the same manner, a solution for the hole transport layer (HTL, poly(N-vinylcarbazole (PVK)) was spin coated onto the PEDOT:PSS at 3000 rpm for 60 s, followed by annealing at 150 °C for 30 min. The QD patterns were sprayed by the EHD jet printer with various parameters and three spraying methods (big circular film, spiral-line, and straight-line methods), followed by drying at room temperature for 10 min. Afterward, a solution for the electron transfer layer (ETL, ZnO) was spin coated at 2500 rpm for 35 s, followed by baking at 110 °C for 30 min.

Finally, a 100-nm of aluminum was deposited on top of the ETL by evaporation at 1.2 × 10^−6^ Torr. Electroluminescence (EL) spectra and current-voltage-luminance (J-V-L) characteristics were examined using a SpectraScan PR 670 Spectroradiometer coupled with a Keithley 2400 source measurement unit. All measurements were recorded under ambient conditions.

## Supplementary information

Optimization of Quantum Dot Thin Films using Electrohydrodynamic Jet Spraying for Solution-Processed Quantum Dot Light-Emitting Diodes
