# Supplementary material for: Optimization of Quantum Dot Thin Films using Electrohydrodynamic Jet Spraying for Solution-Processed Quantum Dot Light-Emitting Diodes
Source: Sci Rep. 2019 Sep 25;9:13885. doi: 10.1038/s41598-019-50181-5 (PMC6761258; doi:10.1038/s41598-019-50181-5)
Supplement: Supplementary file 1 — Optimization of Quantum Dot Thin Films using Electrohydrodynamic Jet Spraying for Solution-Processed Quantum Dot Light-Emitting Diodes [file 41598_2019_50181_MOESM1_ESM.docx]

**Supplementary Information**

**Optimization of Quantum Dot Thin Films using Electrohydrodynamic Jet Spraying for Solution-Processed Quantum Dot Light-Emitting Diodes**

Tuan Canh Nguyen, Thi Thu Thuy Can, and Woon-Seop Choi*


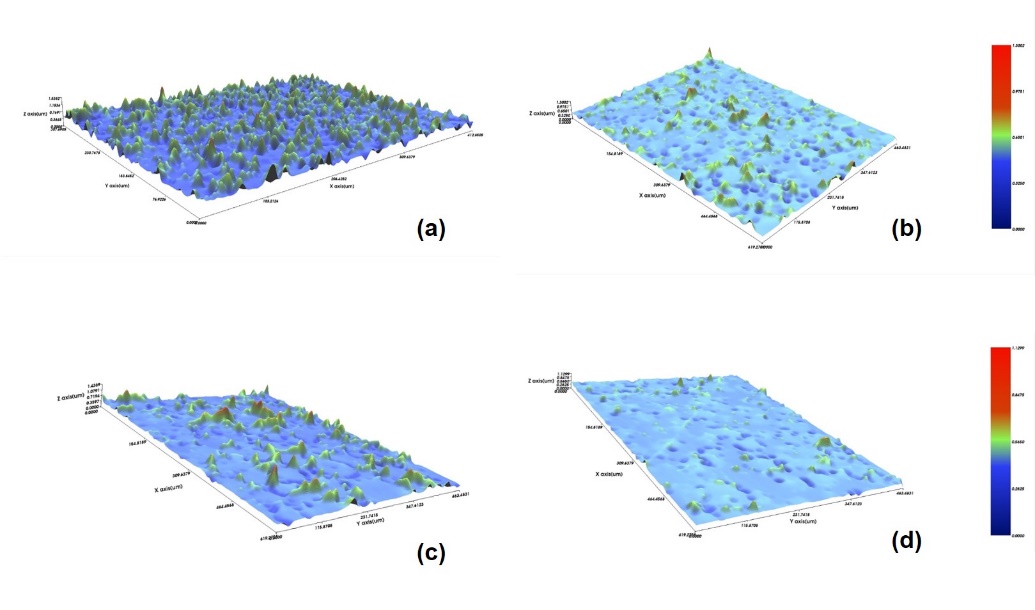


Fig. S1. 3D images of QDs on ITO-glass (NanoSystems) (corresponding to OM images in Fig.5 e-h).


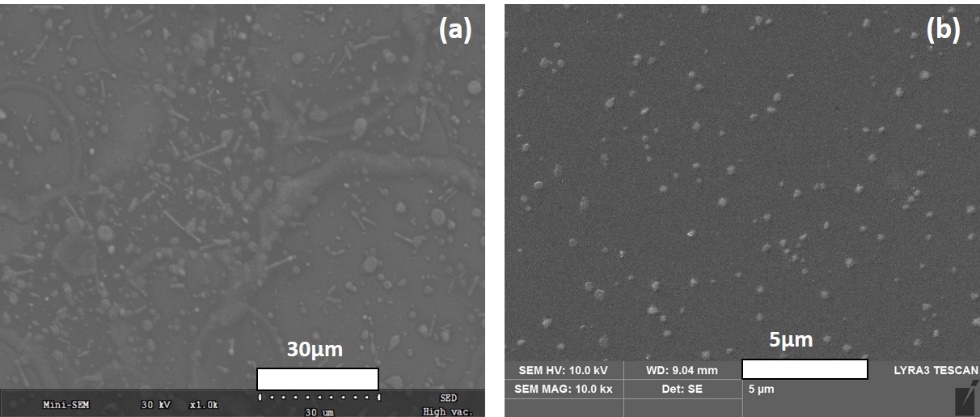


Fig. S2. SEM images of QD-sprayed on ITO-glass using type III spraying method: applied voltage of 3.8 kV, tip height of 12 mm, flow rate of 0.024 µl/sec, and XY stage (2400 µm, 2400 µm).


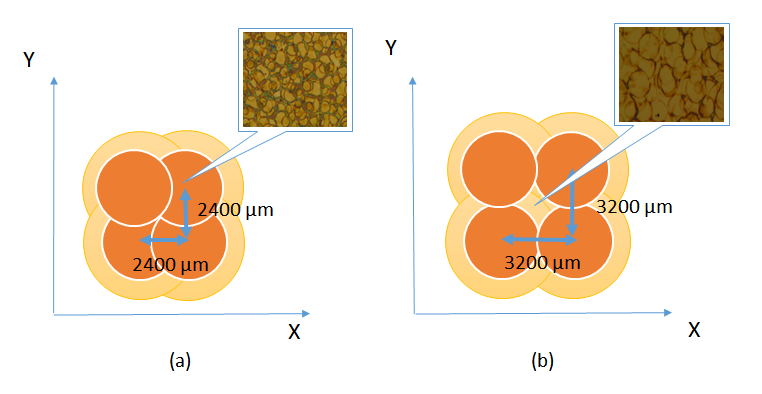


Fig. S3. Two different overlapping modes of droplets with changing X and Y in type III spray method


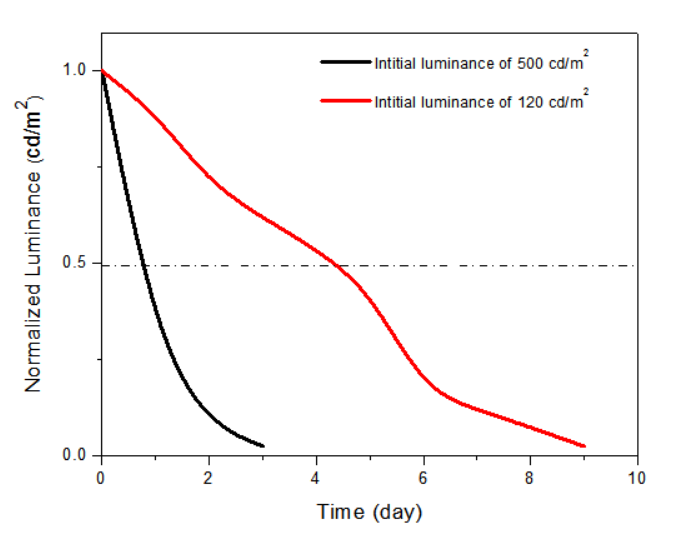


Fig. S4. Life time measurement of QD-LEDs prepared with type III spray method.


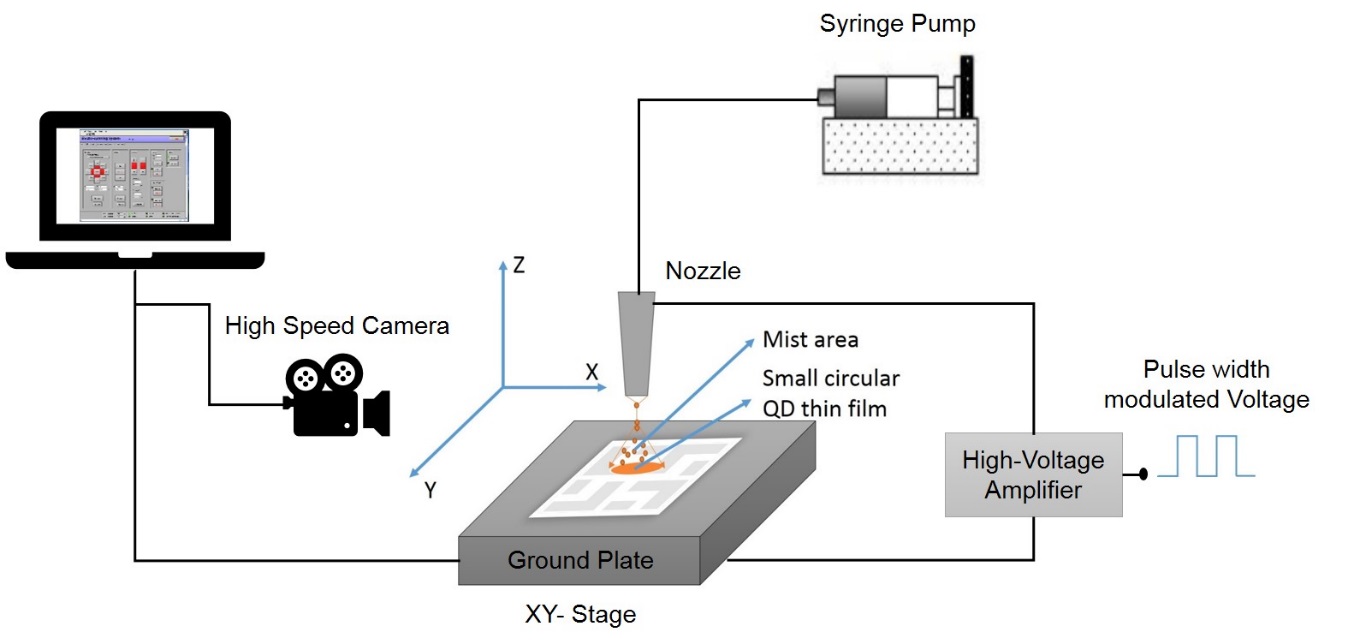


Fig. S5. Diagram of the EHD jet spraying system


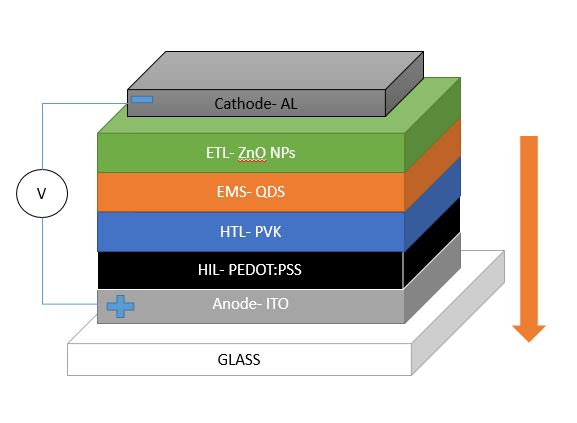
(a)
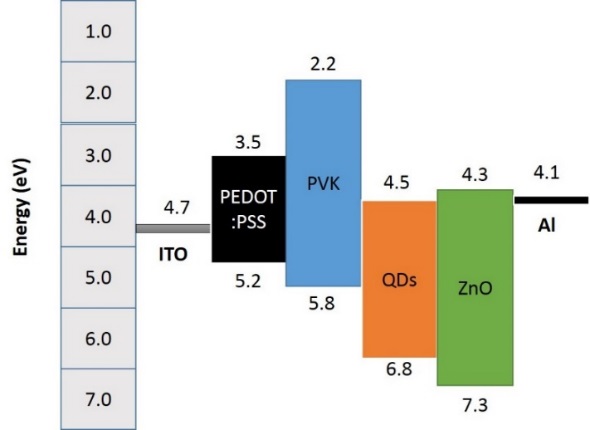
 (b)

Fig. S6 (a) QD-LED device structure and (b) band gap energy diagram of fabricated QD-LED.

Table S1. RMS roughness of QD thin films on ITO-glass (corresponding to Fig. 5e-h) and PVK patterns (corresponding to Fig. 6) using type III spraying method: applied voltage of 3.8 kV, tip height of 12 mm, flow rate of 0.024 µl/sec, and changing XY-stage from 800 to 3200 µm.

| XY-Stage (µm) | RMS roughness R_q_ (µm) | |
| --- | --- | --- |
|  | QD-sprayed on ITO-Glass | QD-sprayed on PVK/PEDOT:PSS/ITO-Glass |
| 800, 800 | 0.1054 | 0.1211 |
| 1600, 1600 | 0.0702 | 0.0902 |
| 2400, 2400 | 0.0492 | 0.0564 |
| 3200, 3200 | 0.0577 | 0.0636 |
